# Protective Effects of Topical Administration of Laminarin in Oxazolone-Induced Atopic Dermatitis-like Skin Lesions

**DOI:** 10.3390/md20110669

**Published:** 2022-10-26

**Authors:** Tae-Kyeong Lee, Dae Won Kim, Ji Hyeon Ahn, Choong-Hyun Lee, Jae-Chul Lee, Soon Sung Lim, Il Jun Kang, Seongkweon Hong, Soo Young Choi, Moo-Ho Won, Joon Ha Park

**Affiliations:** 1 Department of Food Science and Nutrition, Hallym University, Chuncheon 24252, Gangwon, Korea; 2 Department of Biochemistry and Molecular Biology, Research Institute of Oral Sciences, College of Dentistry, Gangnung-Wonju National University, Gangneung 25457, Gangwon, Korea; 3 Department of Physical Therapy, College of Health Science, Youngsan University, Yangsan 50510, Gyeongnam, Korea; 4 Department of Pharmacy, College of Pharmacy, Dankook University, Cheonan 31116, Chungbuk, Korea; 5 Department of Neurobiology, School of Medicine, Kangwon National University, Chuncheon 24341, Gangwon, Korea; 6 Department of Surgery, Kangwon National University Hospital, School of Medicine, Kangwon National University, Chuncheon 24289, Gangwon, Korea; 7 Department of Biomedical Science, Research Institute for Bioscience and Biotechnology, Hallym University, Chuncheon 24252, Gangwon, Korea; 8 Department of Anatomy, College of Korean Medicine, Dongguk University, Gyeongju 38066, Gyeongbuk, Korea

**Keywords:** laminarin, atopic dermatitis, oxazolone, mast cells, immunoglobulin E, proinflammatory cytokines

## Abstract

Laminarin is a polysaccharide isolated from brown marine algae and has a wide range of bioactivities, including immunoregulatory and anti-inflammatory properties. However, the effects of laminarin on atopic dermatitis have not been demonstrated. This study investigated the potential effects of topical administration of laminarin using a Balb/c mouse model of oxazolone-induced atopic dermatitis-like skin lesions. Our results showed that topical administration of laminarin to the ear of the mice improved the severity of the dermatitis, including swelling. Histological analysis revealed that topical laminarin significantly decreased the thickening of the epidermis and dermis and the infiltration of mast cells in the skin lesion. Serum immunoglobulin E levels were also significantly decreased by topical laminarin. Additionally, topical laminarin significantly suppressed protein levels of oxazolone-induced proinflammatory cytokines, such as interleukin-1β, tumor necrosis factor-α, monocyte chemoattractant protein-1, and macrophage inflammatory protein-1α in the skin lesion. These results indicate that topical administration of laminarin can alleviate oxazolone-induced atopic dermatitis by inhibiting hyperproduction of IgE, mast cell infiltration, and expressions of proinflammatory cytokines. Based on these findings, we propose that laminarin can be a useful candidate for the treatment of atopic dermatitis.

## 1. Introduction

Atopic dermatitis (AD) is an inflammatory skin disease presenting with clinical symptoms of severe itching, scratching, edema, and rash [1]. Worldwide, it has a prevalence of 15% to 30% in children and 2% to 10% in adults, leading to a high impairment of quality of life [2,3]. Although the pathogenesis of AD is not completely understood, its involvement has been implicated in the complex interplay among genetic and environmental factors, such as dysregulation of the immune system and dysfunction of the skin barrier [4,5]. The main treatment for AD is topical application of corticosteroids that suppress adverse immune/inflammatory responses [6,7]. Unfortunately, this medication may not be effective and can cause significant side effects, especially in long-term use in children [8,9]. Thus, natural products that can have fewer side effects have received a lot of attention in the treatment of AD [10,11].

**Figure 1:**
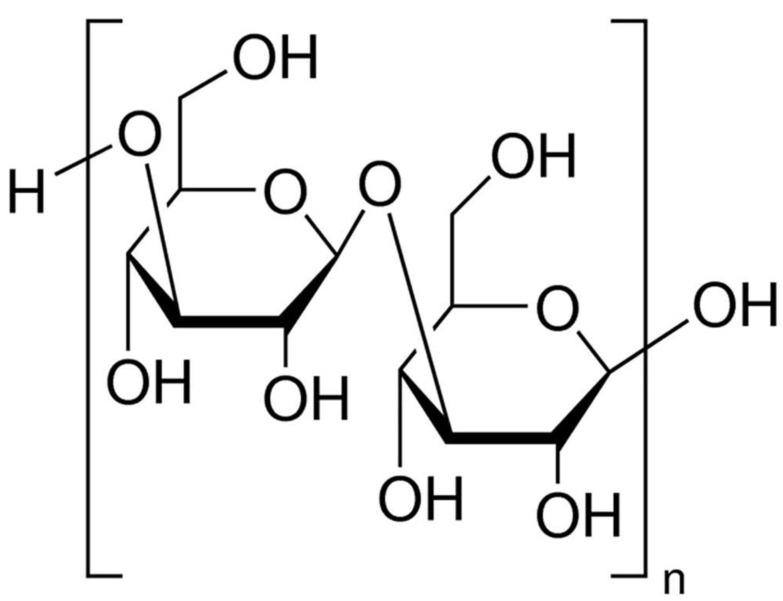
The chemical structure of LA.

Laminarin (LA) is a low-molecular-weight polysaccharide (Figure 1) found in brown marine algae, such as *Laminaria digitata,* and possesses multiple bioactivities, including immunoregulatory and anti-inflammatory properties [12,13,14]. Some studies have shown beneficial effects of topical administration of LA on skin health. For example, topical administration of LA improves the wound healing process in rat skin [15]. In addition, topical application of LA reduces skin damage caused by UVB irradiation in mice [16]. To the best of our knowledge, however, there is no information on the protective effects of LA against AD. Thus, the aim of this study was to examine the protective effects of topical administration of LA in a mouse model of oxazolone (OX)-induced AD-like skin lesions, which has been widely used to assess the therapeutic effects of candidates for the treatment of AD [17,18]. We also investigated whether its effects were performed via anti-inflammatory action of LA in the mouse model.

## 2. Results

### 2.1. Effects of LA on OX-Induced Ear Thickness and Weight

**Figure 2:**
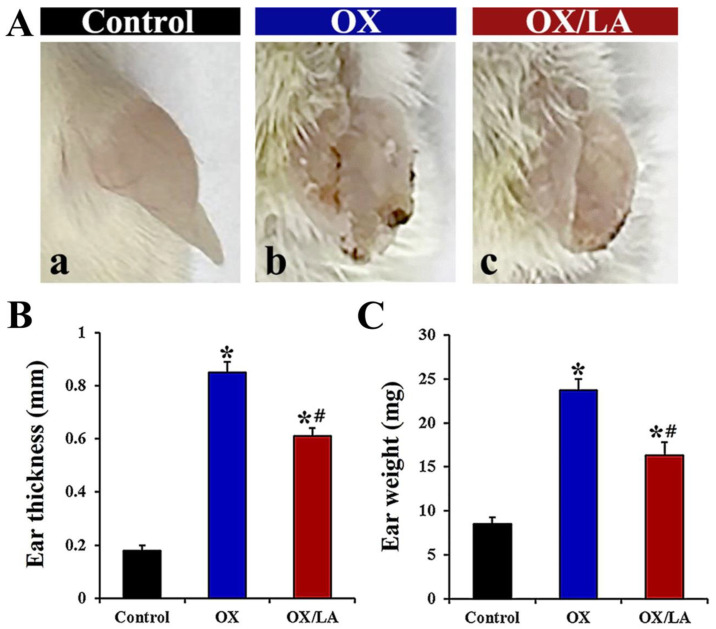
(**A**) Representative photographs of ears from the control (a), OX (b), and OX/LA (c) groups at 28 days after AD induction. In the OX/LA group, the severity of OX-induced AD-like skin lesions was distinctly attenuated compared to that in the OX group (**B**,**C**). Ear thickness (**B**) and weight (**C**) in each group at 28 days after AD induction. In the OX/LA group, ear thickness and weight were significantly decreased compared to those in the OX group. Bars indicate the means ± SEM (*n* = 7, respectively; * *p* < 0.05 vs. control group, ^#^ *p* < 0.05 vs. OX group).

The OX group showed typical symptoms of AD in the ear—such as erythema, cracking, and edema—compared to the control group (Figure 2A(a,b)). In addition, ear thickness and weight in the OX group were significantly increased (approximately 4.7-fold and 2.8-fold, respectively) as compared to those in the control group (Figure 2B,C). In contrast, in the OX/LA group, OX-induced AD-like symptoms were distinctly improved compared to the OX group (Figure 2A(c)), showing that ear thickness and weight were significantly decreased (approximately 28% and 31%, respectively) compared to those in the OX group (Figure 2B,C).

### 2.2. Effects of LA on OX-Induced Changes in Epidermal and Dermal Thickness

**Figure 3:**
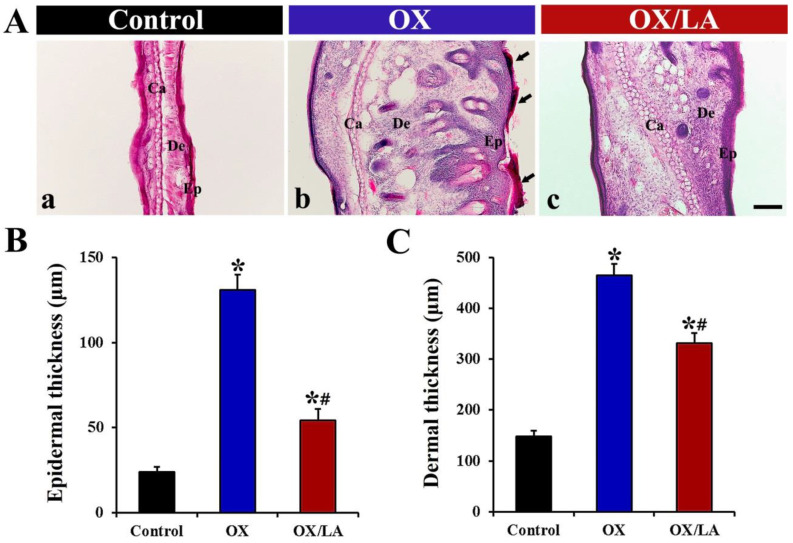
(**A**) Representative images of H&E staining in the ear skin of the control (a), OX (b), and OX/LA (c) groups at 28 days after AD induction. In the OX group, the epidermis and dermis were markedly thickened, and pustule formation (arrows) was observed in the epidermis. In the OX/LA group, OX-induced thickening of the epidermis and dermis was apparently ameliorated. Ca, cartilage; De, dermis; Ep, epidermis. Scale bar = 100 μm. (**B**,**C**) Thicknesses of the epidermis (**B**) and dermis (**C**) in the ear skin of each group at 28 days after AD induction. Bars indicate the means ± SEM (*n* = 7, respectively; * *p* < 0.05 vs. control group, ^#^ *p* < 0.05 vs. OX group).

Changes in thickness of the epidermis and dermis of ear skin, as an index for OX-induced AD-like skin lesions, were examined using hematoxylin and eosin (H&E) staining (Figure 3A). The OX group showed thickening of the epidermis and dermis with pustule formation in the epidermis (Figure 3A(b)). On the other hand, the epidermal and dermal thickening in the OX/LA group was significantly attenuated compared to the OX group (Figure 3A(c)). The thickness of the epidermis in the OX group (131 ± 9.4 μm) was approximately 5.5-fold higher than that in the control group (24 ± 3.2 μm); however, the epidermal thickness in the OX/LA group (54 ± 7.6 μm) was significantly decreased by approximately 59%, compared with that in the OX group (Figure 3B). The thickness of the dermis in the OX group was approximately 465 ± 23.8 μm, which was 3.2-fold thicker than that in the control group (148 ± 11.2 μm), while the dermal thickness in the OX/LA group (332 ± 16.5 μm) was significantly lower (approximately 30%) than that in the OX group (Figure 3C).

### 2.3. Effects of LA on OX-Induced Mast Cell Infiltration and Serum Immunoglobulin E (IgE) Level

**Figure 4:**
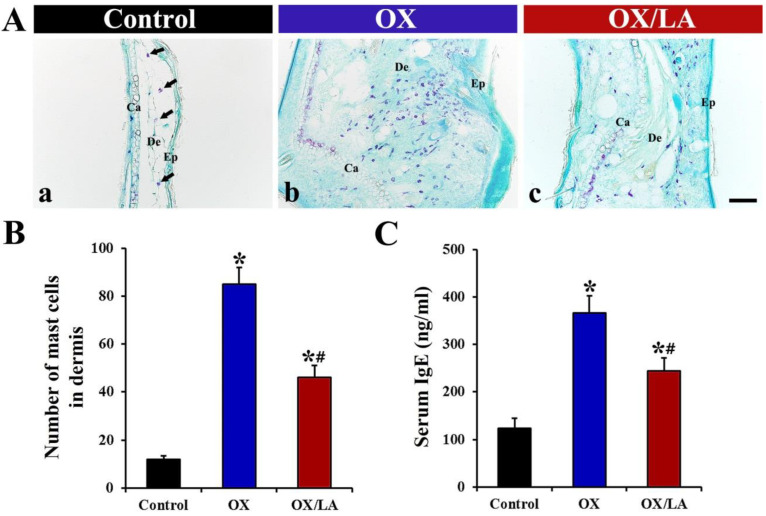
(**A**) Representative images of toluidine blue staining in the ear skin of the control (a), OX (b), and OX/LA (c) groups at 28 days after AD induction. In the control group, a few mast cells (arrows) were found in the dermis. In the OX group, a significant increase in the number of mast cells was observed. However, in the OX/LA group, the number of mast cells was significantly decreased compared to that in the OX group. Ca, cartilage; De, dermis; Ep, epidermis. Scale bar = 100 μm. (**B**) Number of mast cells in the dermis of each group at 28 days after AD induction. Bars indicate the means ± SEM (*n* = 7, respectively; * *p* < 0.05 vs. control group, ^#^ *p* < 0.05 vs. OX group). (**C**) Serum IgE level in each group at 28 days after AD induction. In the OX/LA group, serum IgE levels were significantly lower than those in the OX group. Bars indicate the means ± SEM (*n* = 7, respectively; * *p* < 0.05 vs. control group, ^#^ *p* < 0.05 vs. OX group).

Changes in the infiltrations of mast cells in the ear skin were evaluated by toluidine blue staining (Figure 4A,B). In the control group, a few mast cells (12 ± 1.5 cells), which appear as small purple dots, were observed in the dermis (Figure 4A(a),B). In the OX group, OX-induced mast cells were significantly increased in the dermis compared to those in the control group, showing that the number of the mast cells was 85 ± 7.3 cells (Figure 4A(b),B). However, the number of the mast cells in the OX/LA group (46 ± 5.7 cells) was significantly decreased compared to that in the OX group (Figure 4A(c),B).

Change in serum IgE levels was measured by an ELISA assay kit (Figure 4C). Serum IgE levels in the OX group (367 ± 36.2 ng/mL) were significantly enhanced compared to those in the control group (123 ± 22.1 ng/mL), while serum IgE levels in the OX/LA group (244 ± 28.3 ng/mL) were significantly reduced by approximately 34% compared to those in the OX group (Figure 4C).

### 2.4. Effects of LA on OX-Induced Levels of Proinflammatory Cytokines

**Figure 5:**
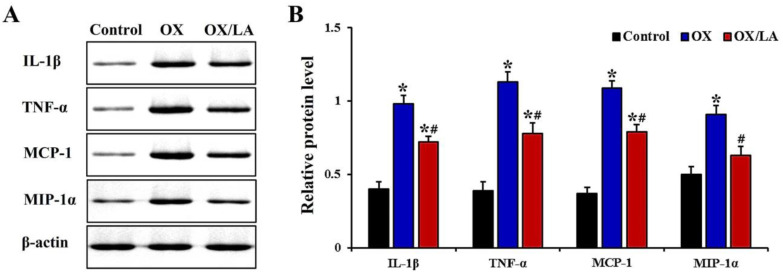
(**A**) Representative Western blot images of IL-1β, TNF-α, MCP-1, and MIP-1α in mouse ear skin of the control, OX, and OX/LA groups at 28 days after AD induction. In the OX group, IL-1β, TNF-α, MCP-1, and MIP-1α levels were significantly higher than those in the control group. In contrast, IL-1β, TNF-α, MCP-1, and MIP-1α levels in the OX/LA group were significantly reduced compared to those in the OX group. (**B**) Quantitative analyses of IL-1β, TNF-α, MCP-1, and MIP-1α levels by normalization to level of β-actin, respectively. Bars indicate the means ± SEM (*n* = 7, respectively; * *p* < 0.05 vs. control group, ^#^ *p* < 0.05 vs. OX group).

Changes in protein expression levels of proinflammatory cytokines—interleukin-1β (IL-1β), tumor necrosis factor α (TNF-α) monocyte chemoattractant protein-1 (MCP-1) and macrophage inflammatory protein-1 α (MIP-1α)—in mouse ear skin were examined by Western blot analysis. In the OX group, the levels of IL-1β, TNF-α, MCP-1, and MIP-1α were significantly increased (approximately 2.5-fold, 2.8-fold, 2.9-fold, and 1.8-fold, respectively) compared to those in the control group. However, the levels of IL-1β, TNF-α, MCP-1 and MIP-1α in the OX/LA group were significantly decreased by approximately 27%, 32%, 28%, and 31%, respectively, compared to those in the OX group (Figure 5A,B).

## 3. Discussion

There is accumulating evidence that shows that brown marine algae-derived polysaccharides have therapeutic potential against diverse skin disorders [19,20]. Among the polysaccharides, fucoidans are found in the cell wall of some brown algae and contain major sulfated polysaccharides (SPs) [21]. Many studies show the beneficial effects of fucoidans for diverse skin disorders including skin aging, atopic dermatitis, and skin carcinogenesis [19]. However, those of LA have been poorly reported to date. LA from the brown algae *Saccharina longicruris* counters skin aging induced by UVA/UVB in Kunming mice, a model of age-dependent decline in female fertility in humans [22,23]. Topical administration of LA from *Cystoseira barbata* in a rat skin wound model heals full-thickness wounds through antibacterial and antioxidant activities [15]. In an in vitro experiment, Ayoub et al. (2015) demonstrated that LA from *Saccharina longicruris* prevented matrix deposition during dermal tissue-engineered production using monolayer-cultured human skin fibroblasts [24].

Here, we first assessed the protective effects of LA on OX-induced AD-like skin lesions in BALB/c mice. In this study, topical administration of OX to the ears of the mice in the OX group successfully induced AD-like skin lesions, which were indicated by typical features such as excoriation, edema, and dryness. Topical administration of LA to the OX/LA group significantly mitigated the severity of the OX-induced AD-like skin lesions. In histopathological examination, epidermal hyperplasia and dermal edema, as typical histopathological signs of AD, were observed in the OX group, whereas these changes were significantly diminished in the OX/LA group. These results indicate that the topical administration of LA may attenuate the symptoms of AD-like skin lesions induced by OX.

In this study, OX-induced mast cells were dramatically increased in the dermis, however, the numbers of the mast cells in the OX/LA group were significantly reduced compared to the OX group. In addition, IgE levels in the serum of the OX group were markedly enhanced, while serum IgE levels in the OX/LA group were significantly decreased compared to the OX group. Mast cells are known to be critical effector cells in IgE-mediated allergic disorders, including AD [25]. Mast cells are activated through cross-linking of allergen-bound IgE to high-affinity IgE receptors, resulting in degranulation of mast cells and release of various proinflammatory cytokines to cause inflammatory responses [26,27]. It was reported that increased serum IgE levels and infiltration of mast cells in AD-like skin lesions were prominent features in AD-like mouse models induced by haptens, such as OX, 2,4-dinitrofluorobenzene (DNFB) and 2,4,6-trinitrochlorobenzene (TNCB) [28,29,30]. Consistent with previous studies described above, our current study showed a significant increase in serum IgE levels and numbers of dermal mast cells in the OX group, while they were significantly decreased in the OX/LA group.

In this study, we found significant increases in protein expression levels of pro-inflammatory cytokines (IL-1β, TNF-α, MCP-1, and MIP-1α) in OX-induced AD-like skin lesions, which were significantly suppressed by topical administration of LA. This finding is particularly important, because pro-inflammatory cytokines are known to play critical roles in the pathogenesis of hapten-induced AD-like skin lesions. For instance, ear swelling due to application of TNCB or OX is strikingly diminished in IL-1β-deficient [31] and TNF-α-deficient mice [32]. In addition, DNFB-induced ear swelling and infiltration of macrophages and T cells are significantly suppressed by administration of short interfering RNA (siRNA) against MCP-1 in mice [33]. In addition, DNFB-induced degranulation of mast cells in mouse skin is significantly inhibited by administration of neutralizing antibodies to MIP-1α [34]. Furthermore, some studies have demonstrated that pharmacological inhibition of IL-1β, TNF-α, MCP-1, and MIP-1α expressions is closely associated with the improvement of OX-induced AD-like skin lesions in mice [29,35,36]. Thus, it seems that topical administration of LA ameliorates OX-induced AD-like skin inflammation by inhibiting IgE hyperproduction, mast cell infiltration, and release of proinflammatory cytokines.

Taken together, our findings suggest that, in mouse ears with OX-induced AD-like skin lesions, topical administration of LA can alleviate AD-like skin lesions by cooperatively inhibiting hyperproduction of IgE and the infiltration of mast cells. Furthermore, the inhibition of IgE hyperproduction and mast cell infiltration by LA may be associated with the suppression of the release of proinflammatory cytokines. Thus, we propose that LA can be utilized as a useful topical agent to assist in improving AD; however, more in-depth studies are necessary to clarify the therapeutic mechanisms of LA.

## 4. Materials and Methods

### 4.1. Animals

Male BALB/c mice (6 weeks old) were purchased from Hana Biotech Inc. (Pyeongtaek, South Korea) and maintained for one week prior to this experiment. The mice were all housed in an air-conditioned room (temperature, 25 ± 5 °C; humidity, 55 ± 5%), and provided with a standard diet and water ad libitum. The experimental protocol for this study, including animal care and handling, was designed in accordance with the guidelines and approval of the Institutional Animal Care and Use Committee of Kangwon National University (approval no. KW-200121-2).

### 4.2. Experimental Groups and Induction of AD-Like Skin Lesions

Mice (total *n* = 21) were randomly distributed to three groups: 1) the control group (*n* = 7), which was exposed to the vehicle; 2) the OX group (*n* = 7), which was exposed to OX alone; and 3) the OX/LA group (*n* = 7), which was exposed to OX in combination with LA.

**Figure 6:**
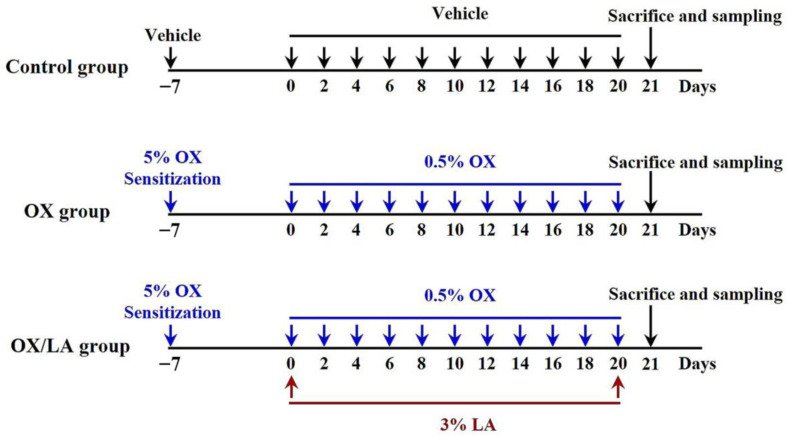
Schematic representation of the experimental schedules to evaluate the effects of LA on OX-induced AD-like skin lesions in mouse ears. The ear skin was sensitized by a single topical treatment of 5% OX. A week later, the ear skin began to be treated with 0.5% OX for three weeks at two-day intervals. At the same time, topical treatment of 3% LA was applied daily for three weeks. On day 21, the mice were sacrificed, and samples were collected for experimental analyses.

OX (Sigma-Aldrich, St. Louis, MO, USA) was dissolved in a vehicle (acetone: olive oil = 4:1 mixture) and used as a sensitizer to induce AD-like skin lesions in mouse ears according to a previously published protocol, with slight modification [37]. In brief, as shown in the Figure 6, the ears were sensitized with 30 µL of 5% OX on the first day of this experiment. After a week of sensitization, 50 µL of 0.5% OX was repeatedly applied to the ears for an additional three weeks at two-day intervals. At the same time, the ears of the OX/LA group were exposed to 50 µL of 3% LA (in distilled water; catalog number, sc-255251; derived from *Laminaria digitata*; purity, ≥96%; Santa Cruz Biotechnology Inc., Santa Cruz, CA, USA) daily for 20 days; the LA application was separated by four hours from that of OX. The dose of LA was determined based on our previous study showing the protective effects of LA against UVB-induced skin damage in mice [16]. The experimental procedure is summarized in Figure 6.

### 4.3. Measurement of Ear Thickness and Weight

At the end of the experiment, the mice were deeply anesthetized by intraperitoneal injection of 200 mg/kg of pentobarbital sodium (JW Pharmaceutical Co., Ltd., Seoul, Korea). To examine the overall degree of the AD-like skin lesions, the ear skins were observed and photographed using a digital camera (Olympus, Tokyo, Japan). After observing the skin lesion, the thickness of the ears was measured using vernier calipers (Mitutoyo, Tokyo, Japan). At sacrifice, blood samples were collected, and ear biopsies (5 mm in diameter) were performed via dermal punch. At the same time, the ears were weighed using a microbalance (Sartorius AG, Göttingen, Germany) and stored in 4% paraformaldehyde solution for histological examination or in a deep freezer for Western blot analysis.

### 4.4. Measurement of IgE

Blood samples were centrifuged at 10,000 rpm for 15 min at 4 °C to obtain serum. Total serum IgE levels were measured using an enzyme-linked immunosorbent assay (ELISA) kit from eBioscience (San Diego, CA, USA) in compliance with the manufacturer’s instructions. Briefly, 96-well plates (Thermo Fisher, Pittsburgh, PA, USA) were coated with a capture-specific antibody for IgE overnight at 4 °C. The plates were washed and blocked with a blocking buffer for two hours at room temperature. Thereafter, serum samples were incubated in appropriate wells of the plates for two hours at room temperature. Biotinylated detection antibody for IgE was added to each well of the plates and incubated for one hour at room temperature. Avidin-horseradish peroxidase (HRP) was continuously added to each well and incubated for 30 min at room temperature. The plates were then washed and incubated with substrate solution for 15 min at room temperature. Finally, stop solution was added to each well, and the absorbance was read at 450 nm.

### 4.5. Tissue Preparation for Histological Examination

Ear tissues were fixed in 4% paraformaldehyde solution according to general procedure. They were dehydrated by serially immersing the tissues in 75%, 80%, 85%, 90%, 95%, and 100% ethanol. They were continuously cleared in graded xylene and embedded in paraffin according to general procedure. Finally, paraffin-embedded tissues were sectioned into eight μm thickness using microtome (Leica, Wezlar, Germany).

### 4.6. H&E Staining

To examine alterations in thickness of the epidermis and dermis of the ears obtained from each group, H&E staining was performed according to a published procedure [38]. In brief, the ear sections were deparaffinized via immersing them in xylene for 20 min at room temperature and hydrated in 100%, 90%, 95%, 80%, and 70% ethyl alcohol for 10 min at room temperature. After that, the sections were stained with Gill’s hematoxylin solution (Merck KGaA, Darmstadt, Germany) for ten minutes at room temperature and briefly soaked in 1% hydrogen chloride (HCl; in 70% ethyl alcohol) followed by 0.5% ammonia (NH_3_; in distilled water). The sections were subsequently stained with eosin Y solution (Merck KGaA, Darmstadt, Germany) for five minutes at room temperature. Next, the sections were dehydrated via serially immersing in ethyl alcohol (50%, 70%, 80%, 90%, 95%, and 100%). Finally, the stained sections were semipermanently preserved by mounting cover glasses with Canada balsam (Kanto Chemical Co., Inc., Tokyo, Japan) as a mounting medium. These prepared slides were examined to measure the thickness of the epidermis and dermis using an Olympus BX53 light microscope (Olympus, Tokyo, Japan) equipped with a DP72 digital camera (Olympus, Tokyo, Japan). The thickness of the epidermis and dermis were measured.

### 4.7. Toluidine Blue Staining

For the detection of mast cells in the dermis, the sections were hydrated as described above and stained with toluidine blue reagent (Sigma-Aldrich) for two minutes. Thereafter, the stained sections were washed, dehydrated, cleared, and mounted with Canada balsam. To examine the change in mast cells, the stained sections were observed with a BX53 light microscope. Digital images of the mast cells were captured, and the mean numbers of the mast cells were counted.

### 4.8. Western Blotting

To examine changes in protein levels of proinflammatory cytokines, Western blot analysis was carried out in accordance with our previous study [39]. Briefly, the ear tissues were ground with a pestle and incubated with 200 µL RIPA buffer containing 50 mM Tris-HCl (pH 8.0), 150 mM NaCl, 0.5% sodium deoxycholate, 1% NP-40, and a protease inhibitor cocktail for one hour on ice. Lysates were obtained by centrifugation, and protein concentration was measured using a bicinchoninic acid protein assay kit from Thermo Fisher (Pittsburgh, PA, USA). An equal amount of lysate was resolved by sulfate-polyacrylamide gel electrophoresis and transferred to nitrocellulose membrane for two hours. The membranes were incubated in 5% skim milk (in PBST; 1X PBS solution supplemented with 0.1% Tween-20) for one hour and hybridized with respective primary antibodies: rabbit anti-IL-1β (diluted 1:1000; Abcam, Cambridge, UK), rabbit anti-TNF-α (diluted 1:1200; Abcam), rabbit anti-MCP-1 (diluted 1:1000; Abcam, Cambridge, UK), rabbit anti-MIP-1α (diluted 1:1000; Abcam, Cambridge, UK), and rabbit anti-β-actin (diluted 1:5000; Sigma-Aldrich, St. Louis, MO, USA) in 1X PBS containing 3% BSA overnight at 4 °C. After washing, the membranes were reacted with HRP-conjugated anti-rabbit IgG (diluted 1:250; Vector Laboratories Inc., Burlingame, CA, USA) for one hour at room temperature and visualized using an enhanced chemiluminescence kit (Pierce Chemical, Dallas, TX, USA).

As described previously [39], the immunoblots of IL-1β, TNF-α, MCP-1, and MIP-1α were analyzed using Scion Image software from Scion Crop (Scion Corp., Frederick, MD, USA). The bands were scanned and densitometric analysis was carried out. The protein levels were normalized versus the corresponding level of β-actin.

### 4.9. Statistical Analysis

All data obtained in this experiment were presented as means ± standard error of the mean (SEM). We performed the Kolmogorov–Smirnov test and Bartlett’s test for evaluation of normal distribution in order to evaluate normal distributions and identical SEMs. Statistical significance was tested by one-way analysis of variance with Tukey’s multiple comparison test. In this study, differences with *p* < 0.05 were statistically significant.

## Data Availability

The data presented in this study are available on request from the corresponding authors.
